# PTSD and crime propensity: Stress systems, brain structures, and the nature of the relationship

**DOI:** 10.1016/j.heliyon.2023.e18381

**Published:** 2023-07-17

**Authors:** Evelyn Svingen

**Affiliations:** Department of Social Policy, Sociology, and Criminology, School of Social Policy, University of Birmingham, Birmingham, UK

**Keywords:** Post-traumatic stress disorder, Crime, Autonomic nervous system, Hypothalamic‒Pituitary‒Adrenal axis, Hippocampus, Amygdala

## Abstract

Post-traumatic stress disorder (PTSD) is the most commonly found disorder among the prison population; however, research has been slow to study it as a potential cause of crime. This review examines the neurophysiological changes in the organism associated with PTSD and connects them to crime and antisocial behaviour. Patients with PTSD suffer from a hyperactive sympathetic nervous system (SNS), an overactive amygdala that results in a hypoactive hypothalamic‒pituitary‒adrenal (HPA) axis, and a reduced hippocampal volume. All these features have been separately associated with aggressivity and antisocial behaviour; however, no consensus has been reached. Moreover, very little research has addressed the need to study the interaction between several stress-response systems. As a result, although there is some indication that patients with PTSD are probabilistically more likely to commit acts of crime, no conclusive results on the influence of PTSD on crime propensity can yet be drawn. Future research should address the interaction between the stress-response systems to understand the nature of antisocial behaviour and violence as well as to study any possible links between PTSD prevalence and possible unrest in prisons.

## 1 Introduction

Post-traumatic stress disorder (PTSD), previously referred to as Vietnam syndrome or shell shock [1,2], has recently gained interest as a topic of research. It was first included in the Diagnostic and Statistical Manual of Mental Disorders (DSM) in 1980 and has been revised several times since then [3]. Consistently having been reported to affect approximately 8% of the European population [[4], [5], [6], [7]], it remains the most commonly found disorder in the prison population [8], especially among women [9,10]. Even though most people do not develop PTSD after a psychological trauma, the effects of PTSD on cognition and behaviour of the individual are multifaceted and impactful, which makes it an important disorder to study.

Even though some of the trauma happens inside prisons, the National Comorbidity Survey suggests that a vast majority of the traumatic events happened to convicted offenders prior to them entering prison and committing the crime for which they were arrested [9,11], which suggests a possible connection between PTSD and offending. However, as a disorder, PTSD has mainly been looked at as a consequence of crime and not necessarily the cause, even though some have started to connect PTSD with offending [12].

As we study both violence in prisons and the relationship between mental health and crime, having an understanding of the neurophysiology of PTSD and its connection (if any) to criminality can provide an invaluable tool in understanding the nature of the disorder for better policymaking and criminological theory. In criminology, there is a significant amount of research examining the relationship between the experience of violence and offending. Exposure to violence has been associated with an elevated risk of delinquency [13], especially child abuse [14,15] and sexual abuse [16].

In this review, I aim to consolidate the neuropsychological and biocriminological evidence existing on the matter by discussing three separate stress-response systems that are affected in the population with PTSD. In doing so, I note that there is some evidence that supports the potential connection between PTSD and crime propensity, but contradictory evidence exists that should be reconciled by future research. In addition, I point out the need for future research that embraces the interactions between the stress-response systems that are often studied separately. Perhaps in contradiction to the convention, in which papers aim to close the gap in the exiting research, this review highlights the current gap and emphasises the need to examine it in further detail.

In this narrative review, I present a literature search of original studies on how PTSD affects various stress-response systems and how that effect can lead to increased criminality from the studies found in MEDLINE, Web of Science, and Google Scholar. I have included studies that examine the neurophysiological mechanisms regulating responses to stress that are consistently found to be dysfunctional in PTSD patients, namely, the hyperactivity of the sympathetic nervous system (SNS), the hypoactivity of the hypothalamic‒pituitary‒adrenal (HPA) axis and reduced hippocampal volume. In addition, I included a search for the connection between the outlined dysfunctions and criminality.

I begin by outlining the summary of the neurophysiology of PTSD and how the different systems fit together. I then proceed to address the sympathetic nervous system (SNS), the hypoactivity of the hypothalamic‒pituitary‒adrenal (HPA) axis and reduced hippocampal volume one by one to address their connection to the existing research on crime propensity.

## 2 Summary overview of the neurophysiology of PTSD

In the close aftermath of exposure to trauma, people who develop PTSD and those who do not express identical symptoms. However, a common response to trauma is resilience: most people go into remission after a month of showing the trauma-related symptoms. A rare percentage, however, does not go into remission and their symptoms remain with them for much longer - this is what we call PTSD [11]. In a rather simplistic view, PTSD is a failed attempt of the organism to adapt to a stressful situation that happened to a person earlier. This attempt is characterised by activating several endocrine and neurotransmitter pathways that activate neurological networks and control both conscious and unconscious responses [17].

People develop PTSD after witnessing, learning about, or experiencing a life-threatening or otherwise dangerous event that could cause bodily harm to oneself or others (criterion A of DSM-V). They suffer from intrusive memories, flashbacks, or thoughts (criterion B); avoidance of memories or reminders of the event (criterion C); changes in mood and cognition (criterion D) and hyperarousal (criterion E) that occur for longer than a month after the trauma (Criterion F; 4). Therefore, there are some observable biological differences that often appear in patients that develop PTSD, and these differences can alter the way the person perceives the world around them and reacts to it.

**Fig. 1:**
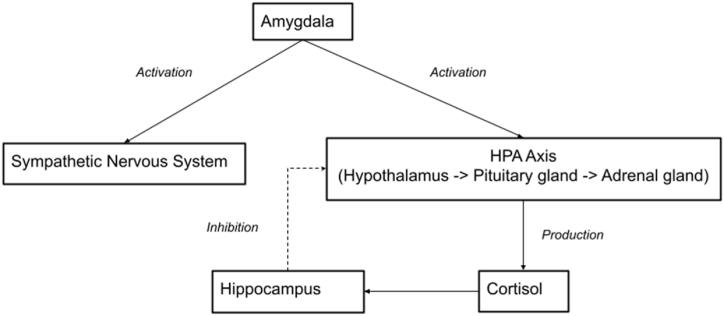
Summary graph of PTSD stress response systems.

Stress response begins with the amygdala - a structure that plays a central role in the processing of emotions, including the one of fear. When the amygdala detects danger, it activates the Sympathetic Nervous System (SNS) and the Hypothalamic-Pituitary-Adrenal (HPA) axis. As HPA axis activates, it releases cortisol, a stress hormone, which, apart from regulating our physiological responses to stress, also activates the hippocampus. The hippocampus, activated by cortisol, works to shut down the working of the HPA axis when its activation is no longer needed. This process is summarised in the diagram below (Fig. 1).

In patients with PTSD, we can normally observe abnormalities in all four structures: the over sensitive amygdala which responds to stress even when the stressors are not there; the faulty workings of the SNS and the HPA axis; and a damaged hippocampus which is not as good at inhibiting the working of the HPA axis and hence does not stop the stress response when it is no longer necessary. The following sections examine the three systems of human stress response system separately.

## 3 Hyperactive sympathetic nervous system (SNS)

PTSD is a failed attempt of the organism to adapt to a stressful situation that happened to a person earlier. This attempt is characterised by activating several endocrine and neurotransmitter pathways that activate neurological networks and control both conscious and unconscious responses [17]. There are several systems of stress response, which are addressed in this and the following section. This section discusses the autonomic nervous system (ANS).

The brain constantly regulates between short-term threat and long-term survival. There are two separate types of ANS responsible for these things: the sympathetic ANS (SNS), the so-called fight-or-flight system, and the parasympathetic ANS (PSNS), the feed-and-breed system. SNS, although being active quite often to sustain basic homeostasis, uses the energy of the body to save itself from an imminent threat. Usually, such a response involves secretion of adrenalin, dilating pupils, increasing heart rate and similar things that save us from danger [18]. The PSNS does exactly the opposite job. It makes the organism store energy by activating processes such as digestion and salivation [19]. These two systems both compete for control at the same time, with one overpowering another depending on the situation. During physical activity, when there is a risk of bodily harm, the SNS is activated, turning the PSNS off. When the harm is away, the opposite happens.

In PTSD patients, SNS is activated even in periods with a relatively safe environment, which is reflected by the diagnostic manual itself by the abovementioned criterion E [4]. From a biological perspective, traumatic events serve as an unconditional stimulus, which, in return, causes unconditioned responses that explain the nature of triggers that may start a panic attack [20]. PTSD damages the balance between sympathetic and parasympathetic responses, which leads to hyperarousal and a fight-or-flight response even in a less stressful situation.

Hyperarousal as such has been connected to many feelings that are traditionally associated with crime. For example, within the sample of victims of violent crime, those who did have PTSD and showed SNS hyperarousal were found to be more easily angered than those without [21,22], as well as expressed stronger feelings for revenge [23]. Higher activation of the SNS has been connected to higher aggression reactivity in children [24], which is further demonstrated by the fact that children with hypertension (also activated by the SNS) have scored higher on the delinquency and aggression questionnaire than their peers [25]. Higher activation of the SNS has also been connected to hostility, antisocial behaviour, and aggressivity in adults [18,26,27]. More importantly, it has been associated with actual crime as well. A study of Vietnam veterans found that SNS hyperarousal was strongly associated with their spouses’ reports of domestic abuse [28]. However, in contrast to these findings, criminological literature has long been connecting a feature of PSNS activity to crime.

Criminological literature is quite consistent at linking aggressivity, crime and antisocial behaviour to a low resting heart rate [[29], [30], [31], [32]], even though it is something that can be commonly found in individuals who exercise a lot. Heart rate is controlled by the ANS, namely, the SNS and PSNS described above. In a resting state, PSNS releases acetylcholine, a hormone that slows the heart rate. When the SNS is activated, it releases hormones (epinephrine and norepinephrine) to accelerate the heart rate. That means that the reduced resting heart rate would depend on the high parasympathetic activity. Not SNS activity, as suggested by the literature above.

Nevertheless, what presents itself as a major contradiction could, in fact, be explained. Low resting heart rate does not automatically mean low SNS activity. It could also point towards hormonal dysregulation in criminals that would not allow for a fight-or-flight reaction to go through or a generally larger heart to the noncriminal population. Considering that this PSNS regulation is socialised in families [33,34], it is also possible that the lower heart rate simply indicates a correlation to adverse parenting. It is also possible that people who are likely to exhibit aggressive behaviours demonstrate both sympathetic underarousal and heightened sympathetic reactivity [18]. For example, males with oppositional defiant disorder (ODD) had both a lower resting heart rate than the control group and a higher heart rate when provoked [35]. A meta-analysis [36] has furthermore suggested that low resting heart rate as well as the low activity of the ANS is connected solely to disruptive behaviour disorder (DBD), which could serve as a confounding factor in studies where it is not controlled for. Unfortunately, in the current state of research, a definite answer to this question cannot be given [36].

Nevertheless, the data suggest that increased activation of the SNS, which is very likely to be induced by PTSD, has been linked to criminality, hostility and aggression. However, these findings are confounded by the criminological literature that links low resting heart rate to crime. The conclusion is even harder to reach when studying the SNS without considering the other systems of stress response, such as the hypothalamic‒pituitary‒adrenal (HPA) axis, to which I turn.

## 4 Hypoactive hypothalamic‒pituitary‒adrenal (HPA) axis

The activation of the hypothalamic‒pituitary‒adrenal (HPA) axis in response to fear starts with an area that is not a part of the axis itself - the amygdala. The amygdala is part of the limbic system and is responsible for regulating emotional input and learning. It registers the adverse consequences of something and then learns to avoid it. By collecting sensory information about a possible stress factor, it activates the hypothalamus, which activates the HPA [19]. Patients with PTSD show increased sensitivity of the amygdala to everyday environments, bringing it to a state of constant alertness as it reacts to an increased number of things as being potentially dangerous [17]. The hyperreactivity of the amygdala has been consistently tied to aggression by itself [37,38], and its hypersensitivity has been found to be made worse by traumatic experiences [39]. In addition to the fact that it is the amygdala that causes the activation of the HPA axis.

The HPA axis is another system of a response to stress. It consists of the hypothalamus, anterior pituitary gland and adrenal cortex [17]. The HPA axis is a major neuroendocrine system involving a complex interaction of three hormone-releasing glands. When the organism is exposed to stress, the hypothalamus, in addition to activating the SNS, activates the HPA axis by secreting corticotropin-releasing hormone (CRH). CRH stimulates the pituitary, and the pituitary releases adrenocorticotropic hormone (ACTH), which stimulates the adrenal cortex. The adrenal cortex then releases glucocorticoids that manage responses to stressors, with the most commonly found glucocorticoid being cortisol. The release of cortisol inhibits both the hypothalamus and the adrenal gland, sending negative feedback to stop the response [19,40].

PTSD has been consistently associated with HPA axis dysregulation. Patients have been found to have their HPA axis in a less responsive, hypoactive state [[41], [42], [43]]. The hypoactivation of the HPA, in turn, has been strongly linked to aggression and conduct disorders as well as antisocial behaviour [[44], [45], [46]]. Studies show that patients with PTSD have higher levels of CRH in their cerebrospinal fluid (CSF) because the receptors of the pituitary are bluntly responsive to CRH [17,[47], [48], [49]]. Thus, the hypothalamus must release more CRH to initiate the reaction in the HPA axis. CRH is believed to regulate fear-motivated behaviours, and using antagonists has been shown to reduce those behaviours [50] as well as show potential to treat anxiety and depression [51].

What it is likely to indicate is that the HPA becomes less responsive to stress factors from the outside, so that a stronger input is needed. That has been connected to criminality as well, although without a firm conclusion. Dempster et al. [52] found that adverse childhood experiences alter the HPA axis activation and as a result led to a higher crime propensity in a way consistent with the mechanisms of PTSD. Cortisol reactivity, on the other hand, has been found to be negatively associated with aggressivity in children by some [35,53] and completely unrelated to antisocial behaviour by others [54]. The contradictions are likely to be explained by the fact that different systems tend to be reactive to different environments; however, future research is needed to investigate the precise contexts, stimuli, and behavioural outputs.

Environments and specific situations become even more important when connecting these findings to the contradictory information from the previous section. The exact role of SNS and HPA axis activation remains a topic for debate; however, there is a certain degree of agreement that it is possible that they are simply responding to different situations [55,56] although they have several shared inputs, which means that they should be activated in response to similar stimuli [57]. There is no relationship between cortisol levels and SNS activation [35], which means that at least to some extent, those systems are separate and the activation of SNS does not mean activation of the HPA.

The interaction of the two systems has been found to explain antisocial behaviour better than each of them individually [58]. Gordis and colleagues [58] found that it was the symmetry of low activations of both that predicted aggressivity, suggesting that asymmetry may be a protective factor. In fact, it was further suggested that high activation of the SNS can be a protective factor against the hypoactive HPA axis [46]. Since PTSD is characterised by hypoactivity in the HPA and hyperactivity of the SNS [59], it seems that individuals with PTSD should exhibit less antisocial behaviour and commit less crime. Unfortunately, there is still not enough research to fully support this conclusion, especially considering the high potential for comorbidity in PTSD patients.

## 5 Decreased hippocampal volume

Prolonged exposure to stress has not only been associated with faulty HPA axis and SNS functioning but also with a decrease in hippocampal volume. Research has not been directed at studying them together. Nevertheless, evidence suggests that both the HPA and hippocampus tend to become damaged together and hence should not be separated [60]. The hippocampus is important for fear conditioning since it correlates long-term and short-term memory by comparing the present and the past. Studies show that prolonged exposure to stress, especially to glucocorticoids that are released in response to stress by the HPA axis, specifically the adrenal gland, may damage its function by impairing dendrite connections and neurogenesis [[61], [62], [63]]. Considering that most people with PTSD do show reduced volumes of the hippocampus and PTSD seems to make it worse [[64], [65], [66]], one can conclude that increased levels of glucocorticoids damaged that area of the brain as a result of a traumatic event in the past.

Studies have consistently shown that when cortisol levels are either above or below the normal range, memory and learning impairments are found [67,68]. The hippocampus is especially important in responding to stress because it is one of the main structures involved in the inhibition of the workings of the HPA axis. Since it is the HPA axis that tends to damage it initially, a hypothesis called the glucocorticoid cascade hypothesis (GCH) has been proposed. GCH argues that excess glucocorticoids (such as cortisol) damage the hippocampus to such an extent that the hippocampus is unable to inhibit the production of glucocorticoids in the future, causing even more glucocorticoids to be released and damaging the hippocampus even more [66].

Decreased hippocampal volume has been previously associated with psychopathy and was claimed to have potential for explaining antisocial behaviour [[69], [70], [71]]. What has been suggested later, however, is that damage to the hippocampus causes affect dysregulation and impairs fear conditioning, which makes the to-be-offender unable to connect crime with punishment or any other negative consequence [72,73]. It is also possible that everything becomes associated with a negative consequence, making it extremely challenging to distinguish. In patients with PTSD, a damaged hippocampus may facilitate associating some things with stress that should not have been associated with it at the first place and make it very hard to distinguish between dangerous and not dangerous stimuli [17]. Such consequences of reduced hippocampal volume have been associated with high impulsivity, resulting in an increased likelihood of serious violent behaviour [74].

## 6 Conclusion

It is important to emphasise that notwithstanding prolonged exposure to stress, many people do not develop PTSD. However, some do. When they do, they suffer damage to all the stress-response systems at the same time. This damage may affect their crime propensity. The first system is the SNS, which activates the fight-or-flight response much more often than it is needed for the survival of an organism. The other is the HPA axis, which becomes much less sensitive. Because of the excess stress, the hippocampus, responsible for the inhibition of the HPA, is reduced in size and functions less well. The amygdala, which collects receptive stimuli and formulates emotions, becomes oversensitive.

Much of the research done with regards to the hippocampus, HPA and SNS and criminality has looked at the three systems separately. However, some relationship with crime has been found in connection to all the systems. Reduced hippocampal volume has been connected to psychopathy, impulsivity and violent behaviour. Hypoactivity of the HPA and hyperactivity of the SNS - antisocial behaviour. The results of some studies suggest that patients with PTSD are more likely to commit crime than the general population; however, no affirmative agreement exists to truly answer this question. It has been suggested that high activation of the SNS can serve as a protective factor against the higher activation of the HPA [46]; however, no evidence on protective factors against the reduced hippocampal volumes can be found. As such, this paper presents evidence for a need to study all three stress-response systems to answer the question of the relationship between PTSD and crime propensity.

This review demonstrates a pressing need for research into the effects of stress on criminality, such as the low resting heart rate versus the diminished parasympathetic cardiac response debate, to explain counterintuitive but not necessarily contradictory results. It is possible that the reason for the contradictory results stems from the fact that the researchers have tended to concentrate on a very specific set of observations instead of looking at the stress-response system as a whole. Future research should also devote more resources to studying the relationship between possible violence and unrest in prisons and the prevalence of PTSD because, as this paper shows, there might be neurophysiological reasons why some prisoners struggle with imprisonment more than others.

As the body of literature studying the relationship between victimisation and crime grows, this paper explains, using PTSD as an example, how the mechanism could partially be neurophysiological in nature.

## Author contribution statement

All authors listed have significantly contributed to the development and the writing of this article.

## Data availability statement

No data was used for the research described in the article.

## Declaration of competing interest

The authors declare that they have no known competing financial interests or personal relationships that could have appeared to influence the work reported in this paper.
